# Variation in the basal immune state and implications for disease

**DOI:** 10.7554/eLife.90091

**Published:** 2024-01-26

**Authors:** Aisha Souquette, Paul G Thomas

**Affiliations:** 1 Department of Immunology, St. Jude Children's Research Hospital Memphis United States; University of Washington United States; École Normale Supérieure - PSL France

**Keywords:** determinants of immunity, immune memory, heterosubtypic, heterologous infection, chronic co-infection, influenza, correlate of protection, correlate of severity

## Abstract

Analysis of pre-existing immunity and its effects on acute infection often focus on memory responses associated with a prior infectious exposure. However, memory responses occur in the context of the overall immune state and leukocytes must interact with their microenvironment and other immune cells. Thus, it is important to also consider non-antigen-specific factors which shape the composite basal state and functional capacity of the immune system, termed here as I_0_ (‘I naught’). In this review, we discuss the determinants of I_0_. Utilizing influenza virus as a model, we then consider the effect of I_0_ on susceptibility to infection and disease severity. Lastly, we outline a mathematical framework and demonstrate how researchers can build and tailor models to specific needs. Understanding how diverse factors uniquely and collectively impact immune competence will provide valuable insights into mechanisms of immune variation, aid in screening for high-risk populations, and promote the development of broadly applicable prophylactic and therapeutic treatments.

## Introduction

### I_0_ - the basal immune state

Baseline and acute immunity significantly vary across the human population. Additionally, numerous studies have identified specific factors that may increase or decrease susceptibility to infection within a population by influencing infection resistance or disease tolerance, but the ability to integrate these factors to develop a rigorous and quantitative understanding of the basal immune profile and its relationship to illness outcome remains elusive.

The connection between baseline immunity and responses to acute infection has largely been explored from the angle of pre-existing immunity. However, establishment of a robust immune response to any given pathogen involves a complex network of immune cell interactions with non-immune cells in the local tissue microenvironment, other leukocytes, downstream signaling, and production of effector proteins. Indeed, studies have shown that prior immune events can shift the state of diverse cell types in barrier tissues, such as parenchymal and stromal cells, thereby establishing tissue inflammatory memory that synergizes with immune cell-mediated memory to enable rapid recall of distinct exposures (Kazer et al., 2023; Ordovas-Montanes et al., 2020). A variable that alters any component of this system has the potential to change the magnitude or quality of the immune response and, subsequently, disease outcome. Thus, in assessing immunity during infection or response to vaccination, we need to consider the composite basal immune state as the context in which pathogen-specific responses occur and the extent to which this varies, as it may reflect distinct immune profiles that require unique approaches for effective treatment. This pre-existing immune state of an individual, referred to here as I_0_ (‘I naught’), varies across individuals and has important implications for immune competence, as it reflects the functional capacity of a given immune profile. In addition to the magnitude and quality of pre-existing, antigen-specific immunity, I_0_ accounts for non-pathogen-specific factors that can modulate an immune response to challenge, such as the microenvironment (e.g. cytokines) and poised state of myeloid and tissue-specific cells (e.g. basal transcription of pathogen recognition receptors or alarmins).

In this review, we outline our current understanding of the I_0_ landscape, review its determinants, discuss how this relates to susceptibility to infection and/or severe disease, and outline a mathematical framework on how to incorporate I_0_ into future research. It is important to note that the effects of these factors are context dependent. Thus, in order to illustrate the relationship between I_0_ and acute immunity with concrete examples, we have chosen to focus on influenza virus infection, as this infection has been well studied in the context of each factor to be discussed.

### Modeling immunity

Animal models are an invaluable tool in the biological sciences, in part because they allow investigators to fix many variables which could alter an outcome of interest and that cannot be controlled in an analogous human study. However, these models cannot adequately represent the complexity of the human populations we aim to treat, and this contributes to the difficulty in translating scientific findings from the bench to the clinic. Although human studies are more easily translatable, they are often more challenging due to the complicated network of factors that influence disease onset and prognosis, and the substantial human to human variation in said factors. Researchers often attempt to address this via reductive scientific approaches. Population complexity can be simplified via study design through inclusion and exclusion criteria for participant recruitment. Additionally, the influence of a variable can be tested by stratifying groups prior to a statistical analysis. While effective and informative, these reductive approaches can lead to inconsistent results across human studies and mask important underlying biological mechanisms, thereby delaying progress in therapeutic development and/or resulting in poorly efficacious treatments when applied to broader populations in clinical trials.

To demonstrate the relevance of this issue in the context of human immunity - half of 12 prominent studies regarding human immune heterogeneity state recruitment was intentionally restricted to one sex or a given age range, otherwise variability that is attributed to a determinant category they are uninterested in would increase, thereby decreasing power (Brodin et al., 2015; Carr et al., 2016; Lakshmikanth et al., 2020; Lee et al., 2014; O’Neill et al., 2021; Orrù et al., 2013; Patin et al., 2018; Raj et al., 2014; Randolph et al., 2021; Roederer et al., 2015; Tsang et al., 2014; Ye et al., 2014). While this is a valid approach to focus on the effects of a particular determinant of immune heterogeneity, this can severely underestimate the scope of immune variation, mask important immunological signatures and how determinants interact with each other, observed associations cannot be assumed to broadly apply to other demographic populations, and results across studies are often contradicting. While likely unintentional, inclusion and adequate representation (for statistical purposes) of diverse ancestries is often, if not the most, lacking demographic feature in human studies. This presents the same issue as intentional recruitment restrictions, is pervasive across many fields of human research, and significantly contributes to health disparities.

Improving our understanding of human immunity and the implementation of knowledge gained will require more complex study populations that are better reflective of the diverse populations we aim to treat. The analytical approach should be less dependent on reductive techniques, and instead utilize a more comprehensive assessment of multiple factors simultaneously, such as multivariable statistical modeling, in order to account for the intricate network of factors present, and better understand the collective influence and relative importance for a given outcome of interest. The insight gained from such studies can be further utilized to identify optimum points of intervention to mold immunity toward a desired set point to prevent disease occurrence or severity.

**Figure 1.**
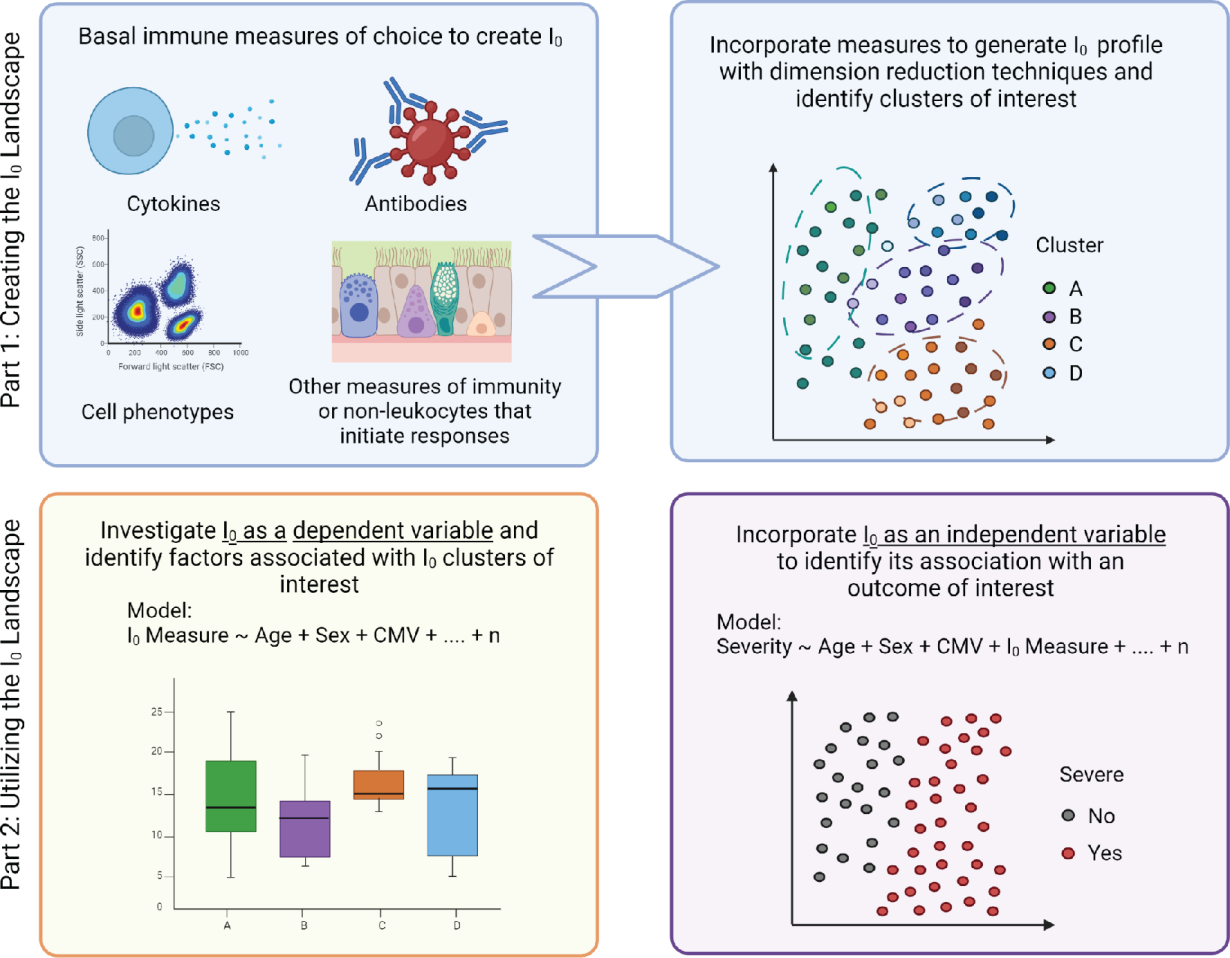
Modeling immunity. Overview of the experimental, conceptual, and mathematical framework of I_0_ (detailed in the ‘Implementing I_0_’ section).

In the mathematical framework sections, we outline how to create an I_0_ landscape, and, utilizing multivariable statistical modeling, how to investigate its determinants, and utilize it as a predictor for the magnitude and quality of an acute immune response and/or illness outcome (Figure 1). Importantly, the quality of the model outputs is dependent on good quality metadata, analytical and experimental approaches. Therefore, we also propose a set of initial ‘core independent factors’ for studies of infectious disease immunity, and discuss how to optimize model inputs and across study comparisons with more consistent metadata collection, comprehensive experimental approaches, high-resolution data, and diverse study populations.

### Determinants of I_0_

**Figure 2.**
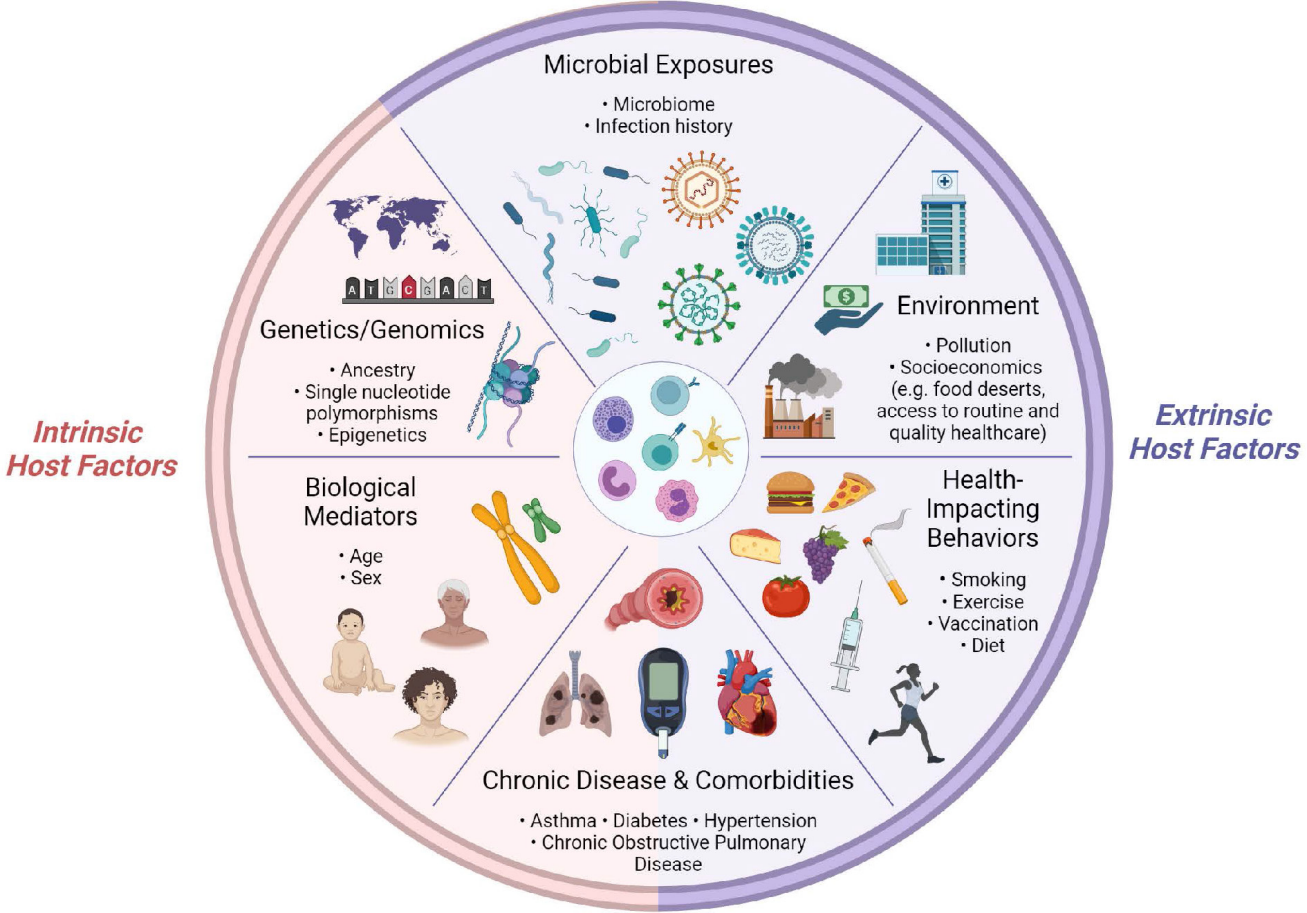
Determinants of immunity. Summary of intrinsic and extrinsic host factors which contribute to immune heterogeneity. Bullet points highlight examples of each determinant.

The immune system, at the I_0_ state and in response to challenge, is shaped by dynamic interactions between intrinsic and extrinsic host factors (Figure 2). Intrinsic factors act from within an individual and include: (1) biological mediators, such as age and sex; (2) genetic variables, such as ancestry; (3) predisposition to chronic disease and/or comorbidities, such as type I diabetes. Extrinsic factors are external to the host and include: (1) health-impacting behaviors, such as vaccination and smoking; (2) non-constitutional, extrinsic environmental factors, such as socioeconomic status, which can impact access to healthy foods and medical care; (3) microbial exposures; (4) chronic disease and/or comorbidities which can be driven by external factors, such as obesity. Note, chronic medical conditions are listed twice, as they are complex diseases which often arise from a combination of genetic (intrinsic) and environmental (extrinsic) factors, but for the purposes of organization in this section, we have included them once under the ‘Extrinsic host factors’ subsection.

For the purposes of this review, we have selected two examples of intrinsic and extrinsic host factors to discuss in further detail, based on two criteria: (1) the breadth of research regarding their effects on I_0_ and/or acute responses to influenza virus infection and (2) whether a factor was included in the set of ‘core independent factors’ (detailed in the ‘Implementing I_0_’ section).

### Intrinsic host factors

#### Age

Extremes of age are a significant risk factor for severe influenza virus infection. Children under 5 years of age, especially infants under the age of 2, are at higher risk of complications (Clohisey and Baillie, 2019; Ruf and Knuf, 2014). This is thought to be due, at least in part, to lack of pre-existing immunity and a heavy reliance on CD4 type 2 responses in children as opposed to the CD4 type 1 and CD8 T cell predominant responses in adults (Cerwenka et al., 1999; Chang et al., 2011; de Kleer et al., 2016; Dowling and Levy, 2014; Duan and Thomas, 2016). While more studies are needed to fully understand the infant immune system, recent studies have provided insight into their unique immune responses. For example, a study of naturally acquired influenza infection in humans found that, compared to adults, infants and young children exhibit a hyperinflammatory cytokine profile (Oshansky et al., 2014).

For adults, severe influenza infection is most prevalent in those greater than 65 years of age (Gounder and Boon, 2019). This is thought to be largely due to immunosenescence - a set of age-related changes that affect both innate and adaptive immune compartments. Changes include altered extracellular microenvironments, soluble factors important for leukocyte homeostasis and differentiation, and modified immune cell phenotype and functional profiles. Collectively, these changes result in dramatic impairment of immune function, leaving older adults more susceptible to infectious diseases (Bulut et al., 2020; Cunha et al., 2020; Keilich et al., 2019; Molony et al., 2018; Moreau et al., 2017; Shaw et al., 2013). General changes in innate immune cells include, but are not limited to, reduced chemotaxis, phagocytosis, superoxide production, receptor signal transduction, antigen presentation, and interferon production (Feng et al., 2021; Keilich et al., 2019; Molony et al., 2018). Indeed, studies of dendritic cells (DCs) from elderly donors show impaired type I and III interferon production in response to stimulation with influenza virus (Prakash et al., 2013; Sridharan et al., 2011). This reduction was associated with decreased phosphorylation of interferon regulatory transcription factor 7 (IRF7), decreased T cell proliferation, and CD8 T cell effector function, as measured by perforin and granzyme production (Sridharan et al., 2011).

General age-associated changes in adaptive immunity include decreased B cells and antibody diversity, impaired regulatory T cell function, decreased naive T cell numbers concomitant with increased memory T cells, and decreased expression of receptors important for T cell activation and/or differentiation (Britanova et al., 2014; Frasca et al., 2004; Fulop et al., 2017; Keilich et al., 2019). For example, studies of influenza-specific CD8 T cells from older donors exhibited reduced diversity in the T cell receptor (TCR) repertoire, suggesting increased risk for antigenic escape (Gil et al., 2015; Naumov et al., 2008).

At least one hallmark of immunosenescence, the decrease in the naive CD8 T cell population and increase in differentiated memory CD8 T cells, may also be affected by confounding factors. Age-related biological changes that impact these subsets include thymic involution and decreased output of lymphoid lineage committed hematopoietic stem cells (Beerman et al., 2010; den Braber et al., 2012; Elyahu and Monsonego, 2021; Palmer et al., 2018; Pang et al., 2011; Thomas et al., 2020). Chronic infections also contribute to this imbalance, with the most well-known effect from cytomegalovirus (CMV)-induced ‘memory inflation’, which results in large expansions of CMV-specific memory T cells over time (Adler and Reddehase, 2019; Griessl et al., 2021; Whiting et al., 2015; Zangger and Oxenius, 2022). Further highlighting the significance of CMV infection in shaping the CD8 T cell compartment, one study found aging alone, in the absence of CMV infection, does not augment memory T cell numbers in the periphery (Wertheimer et al., 2014). In this context, CMV is an external factor that synergistically interacts with age and augments its apparent effect on the T cell compartment.

The findings presented here exemplify the complex effects age can have on the immune system and how additional factors may modulate that effect. In the context of I_0_ and influenza infection, these data suggest that the response trajectories of the immune system associated with extremes of age are distinct and opposing, such that pediatric subjects are poised for hyperresponsiveness, whereas the elderly for hyporesponsiveness - both can present a risk to the host through excessive cellular damage and impaired tissue integrity, though the former is mediated by immunopathology and the latter is damage associated with uncontrolled pathogen growth.

#### Genetics

Human studies have demonstrated variation in immunophenotypes during health and immune challenge based on ancestry, with important implications for population differences in susceptibility to infection and illness outcome (Mangino et al., 2017; O’Neill et al., 2021; Orrù et al., 2013; Randolph et al., 2021). Identification of human genes that are key during anti-influenza immunity have occurred through studies of inborn errors of immunity and population genetics (Casanova and Abel, 2022; Clohisey and Baillie, 2019).

Primary immunodeficiencies that arise from inborn errors are generally rare, often present in childhood, and have a deleterious effect on protein expression and/or function. An example of this was recently shown in Ciancanelli et al. where an otherwise healthy child presented with severe acute respiratory distress syndrome during primary influenza infection. The child’s parents were heterozygous for two different loss-of-function mutations in the IRF7 transcription factor, thus they were able to produce enough *IRF7* to prevent severe infection; however, the child inherited both mutations which led to a complete loss of functional *IRF7* (Ciancanelli et al., 2015). Activated IRF7 leads to expression of type I and III interferons, and downstream effects are important in establishing antiviral immunity. Coinciding with this, the patient’s leukocytes showed impaired production of type I and III interferons in response to influenza virus, and immortalized fibroblasts from the patient showed 2-log higher influenza virus titers at 48 hr post infection relative to healthy controls (Ciancanelli et al., 2015). Recent studies have also identified associations between severe influenza pneumonitis and immunodeficiencies in IRF9 and TLR3, further highlighting the significant impact of inborn errors of immunity in genes related to the initiation or transduction of interferon responses during influenza infection (Hernandez et al., 2018; Lim et al., 2019).

More common are genetic variants which modulate, as opposed to abrogate, protein expression or function. Related to infection, these mutations can occur in proteins important for viral entry, virus sensing, downstream signaling once a virus is detected, transcription factors which initiate antiviral immunity, cytokines which aid in mediating immune responses, antiviral restriction factors, antigen presentation for adaptive immunity, and factors important for cell homeostasis and/or differentiation upon activation (Kenney et al., 2017). Recent studies of single nucleotide polymorphisms (SNPs) in the interferon-induced transmembrane protein 3 (*IFITM3*) gene demonstrate the potential impact of such variants in the susceptibility to severe influenza infection. *IFITM3* is an interferon-stimulated gene which has been shown to function as a viral restriction factor by blocking virus-host membrane fusion and augmenting antibody-mediated neutralization of influenza A virus (Brass et al., 2009; Desai et al., 2014; Gorman et al., 2016; Lanz et al., 2021). In populations of Asian descent, SNP rs12252-C has consistently been associated with severe influenza illness; however, studies in populations of European descent have shown low prevalence of the risk allele and mixed results regarding disease severity (Everitt et al., 2012; Yang et al., 2015; Zhang et al., 2013). In a study of three independent influenza cohorts, *IFITM3* SNP rs34481144-A was enriched in severe patients and prevalent in European populations (Allen et al., 2017). This SNP is located within the promoter region of *IFITM3,* and the risk allele is associated with decreased expression of *IFITM3* and disrupted transcriptional correlations between *IFITM3* and its neighboring genes. Results from luciferase reporter assays in HEK293T cells and using plasmids with a minimal promoter show that rs34481144 alters promoter activity and modulates reporter gene expression at baseline and in response to stimulation with poly(I:C), live influenza virus, and interferon alpha. Importantly, this study also found that the risk genotype of rs12252 is always inherited with the protective genotype of rs34481144, suggesting the risk alleles are on opposite haplotypes and have independent mechanisms (Allen et al., 2017).

Collectively, these data show there are strong genetic associations with immune outcome, and even in these genetic associations, there are significant ancestral differences likely causing variation in response to infection or disease across diverse populations. Genetic variants that augment antiviral activity or limit proviral factors would result in a protective I_0_ profile, the converse would result in an I_0_ profile associated with infection or severe disease. The *IFITM3* SNPs discussed here demonstrate impairment of antiviral function, and thus contribute to a susceptible I_0_ profile. Additional examples of genetic determinants of immunity can be found in two recent reviews (Gounder and Boon, 2019; Kenney et al., 2017).

### Extrinsic host factors

#### Chronic disease and comorbidities

Classical inflammation, induced by tissue injury or acute infection, is high in magnitude and transient in nature. More recently described, para-inflammation arises from cellular stress or tissue malfunction, and is characterized by systemic low-grade inflammation, which alters homeostatic set points, and promotes the development of chronic inflammatory diseases (Medzhitov, 2008). A primary driver of para-inflammation is a dysmetabolic microenvironment that is driven, at least in part, by comorbidities such as obesity, dyslipidemia, hyperglycemia, and hypertension. These chronic medical conditions are often co-occurring, have been associated with poor outcome to immune challenge, and contribute to the inflammatory state that drives the overt insulin resistance that is central to the etiopathology of metabolic syndrome (Medzhitov, 2008; Paragh et al., 2014; Zmora et al., 2017).

In the context of infectious diseases, there are two main mechanisms for altered illness outcomes. The first is through augmented pathogen fitness and virulence (Honce et al., 2020; Hostetter, 1990). For example, in mouse models of influenza infection, obese mice exhibit increased viral spread and diversity in virus quasispecies (Honce et al., 2020). Additionally, obese host-passaged influenza viruses showed higher mutations associated with virulence and increased replication kinetics in vitro. Coinciding with these results, differentiated normal human bronchial epithelial cells from obese hosts exhibited increased viral replication and impaired interferon responses upon infection with H1N1 influenza virus (Honce et al., 2020).

The second mechanism is through lower magnitude and quality of immune responses, independent of viral factors. Mouse and human studies have shown that diabetics have impairments in multiple facets of innate immunity, such as in leukocyte recruitment, neutrophil reactive oxygen species production, NK cell activating receptor signaling, and monocyte/macrophage phagocytosis and cytokine production (Berbudi et al., 2020; Geerlings and Hoepelman, 1999). Such reduced innate immune function could ultimately limit adaptive immunity through reduced recruitment and co-stimulation of lymphocytes. In support of this, impairment of T cell responses during metabolic stress has been shown for viral challenge, wherein peripheral blood mononuclear cells from obese individuals showed reduced activation of CD8 T cells and production of effector molecules in response to stimulation or vaccination with influenza virus (Paich et al., 2013; Sheridan et al., 2012). Moreover, a recent study in mice has demonstrated an additional mechanism by which chronic condition-associated alterations in innate cell function can impact adaptive T cell responses - modified peptide processing and antigen presentation. Glycation and glycoxidation are post-translational modifications (PTMs) capable of generating neo-epitopes and modifying protein-protein interactions (Clement et al., 2021). Hyperglycemic and hyperlipidemia conditions favor these reactions, as they are non-enzymatic and the rate at which they occur depends on metabolite availability. Indeed, the antigen presentation machinery and peptidome of DCs from obese mice exhibit unique and increased oxidative PTMs, resulting in epitope-specific alterations in peptide presentation (Clement et al., 2021). These studies highlight how chronic conditions can induce a dysmetabolic microenvironment that shapes I_0_ and can ultimately culminate in impaired immunity.

#### Infectious exposures and pre-existing immunity

Prior infectious exposures can modulate tissue and immune compartments, resulting in altered functional responses upon subsequent homologous or heterologous infection and vaccination (Kazer et al., 2023; Sparks et al., 2023). Immune memory, the capability of the immune system to respond more rapidly and robustly upon secondary infection, was traditionally thought to be a unique characteristic of the adaptive immune compartment. Although there is a growing number of studies which demonstrate that innate immune cells also exhibit altered functional profiles after the return to a non-activated state, including after influenza virus infection, there has been limited investigation of its impact on influenza illness outcome (Aegerter et al., 2020; Barton et al., 2007; Bekkering et al., 2021; Bekkering et al., 2014; Chen et al., 2014; Ciarlo et al., 2020; Netea et al., 2020; Quintin et al., 2012; Saeed et al., 2014; Wimmers et al., 2021). Thus, this section will focus on the effects of infectious exposures on adaptive immune memory.

Protection conferred by B cells is humoral-mediated, antibodies (secreted versions of the B cell receptor) circulate throughout the body, and serve neutralizing and non-neutralizing functions that defend against influenza infection and/or severe disease (Henry Dunand et al., 2016; Kim et al., 2016; Krammer, 2019; Ng et al., 2019; Rajendran et al., 2021; Tan et al., 2016). However, immunodominant targets of anti-influenza humoral immunity are often sites of mutation, which can lead to immune evasion (Krammer, 2019; Wu and Wilson, 2017). Given influenza virus’ high rate of mutation, this makes it difficult to target via vaccination, thus prophylactic strategies have begun to focus on inducing broadly reactive antibodies, capable of recognizing multiple strains of influenza virus (Krammer, 2019; Sangesland et al., 2019). One challenge to the development of such vaccines is pre-existing anti-influenza humoral immunity, as most individuals acquire influenza virus multiple times throughout their lifetime and this can alter the breadth and quality of antibody responses (Neu et al., 2016).

Original antigenic sin refers to the concept that the first exposure to influenza virus infection leaves an immunological imprint that conditions immunity to subsequent influenza challenge (Francis, 1960; Zhang et al., 2019). Research has identified and refined the different facets of original antigenic sin, including primary addiction, epitope masking, and antigenic seniority/imprinting (Krammer et al., 2018; Schiepers et al., 2023). Primary addiction refers to the active suppression of de novo B cell responses by pre-existing immunity, the effects of which have been shown to be dependent on antigenic distance, such that it decreases as antigenic distance increases between priming and boosting strains (Schiepers et al., 2023). Epitope masking occurs when pre-existing antibodies block or sterically hinder access to antigen, thereby blunting the adaptive response due to reduced antigenic load (Henry et al., 2018). Antigenic seniority is a model in which the first influenza strain an individual is exposed to takes a ‘senior’ antigenic position in the humoral response, and each subsequent influenza infection is progressively ‘junior’ (Henry et al., 2018). This observation is mediated, at least in part, by ‘back-boosting’, wherein each sequential encounter with influenza boosts the antibody response to prior influenza exposures; thus, the most senior influenza strain is associated with the highest antibody titer due to the most boosting events (Henry et al., 2018). Imprinting may be detrimental if the resulting antibody repertoire is too narrow, as this provides increased opportunity for antigenic escape variants (Zhang et al., 2019). However, recent studies suggest that antigenic imprinting may also be beneficial and can provide protection against novel strains, if the emerging influenza strain is within the same HA subtype as the first imprinting strain (Gostic et al., 2019; Gostic et al., 2016). Thus, shifts in the dominant HA subtypes circulating over time may underlie variation in susceptibility to novel strains in different age groups, thereby contributing to a protective or risk I_0_ profile if the HA subtype is a match or mismatch to the imprinting strain, respectively.

Imprinting effects from primary exposure that influence secondary infection immunity and illness outcome have also been observed in the T cell compartment. In contrast to B cells, T cells often target internal, conserved proteins, and thus are a strategic advantage in the establishment of heterosubtypic immunity, as their cognate antigen is likely to be very similar, even across antigenically distinct strains of influenza. In addition, cross-reactive T cells are capable of recognizing variations of a given epitope. Studies in C57BL/6 mice have shown that mice primed with H9N2 or H1N1 are protected against secondary challenge with novel H7N9 and exhibit early infiltration of CD8 T cells, reduced morbidity and mortality, pulmonary viral load, and time to viral clearance (Duan et al., 2015). Moreover, the size of the memory CD8 T cell response was a predictor of positive outcome and modifying the size of the memory CD8 T cell pool modulated its protective effects, highlighting the important role of CD8s in conferring heterosubtypic protection (Duan et al., 2015). In the influenza mouse model, CD8 T cells target PB1 (polymerase basic protein 1), PA (polymerase acidic protein), and NP (nucleoprotein). Across the three influenza strains used in these studies, these three epitopes were either conserved or contained one to two mutations. Although both H9N2 and H1N1 ultimately conferred protection, they induced different CD8 T cell immunodominance hierarchies and differed in the degree to which they elicited beneficial outcomes (Duan et al., 2015). These results demonstrate how variation in initial influenza virus exposure, a common occurrence in humans, can alter I_0_ by inducing memory T cell responses that vary in magnitude and/or quality, and which can lead to differential outcome upon secondary infection. A memory T cell pool that is smaller in size, non-cross-reactive, narrow in repertoire diversity, or inappropriately targeted (with respect to immunodominance hierarchy) will contribute to a risk I_0_ profile. However, the memory T cell pool, and thus I_0_, can be tailored to confer optimal protection, provided the appropriate priming strain is selected. This underscores the need for further investigation into the identification of strain(s) and strategies that will induce an I_0_ profile associated with optimum protection against current and emerging strains of concern.

Resident memory T cells (T_RM_) are a special subset of T cells that are long-lived, reside in tissues without recirculation, and can confer both antigen-dependent and -independent mechanisms of protection against homologous and heterologous pathogenic challenge. Specifically, mouse studies have shown that T_RM_ cells produce antiviral cytokines more rapidly than systemic effector memory T cells, can quickly trigger innate and adaptive immune responses, augment maturation of local DCs and NK cells, and are capable of inducing broadly active antiviral and antibacterial gene expression, thereby inducing a tissue-wide ‘pathogen alert’ and effectively acting as alarmins (Ariotti et al., 2014; Jiang et al., 2012; McMaster et al., 2015; Pizzolla et al., 2017; Schenkel et al., 2014). Of note, inclusion of T_RM_ into I_0_ calculations (discussed in ‘Implementing I_0_’ section) will be limited by the difficulty in obtaining human tissue samples, as opposed to blood, which is easily collected and less invasive. However, in unique experimental designs for which this is possible, T_RM_ would likely be enriched in protective I_0_ profiles for respiratory infections through rapid initiation of immunity and augmented viral clearance.

In addition to TCR signaling upon antigen recognition (signal 1), co-stimulation by antigen-presenting cells (APCs) and cytokines in the microenvironment provide signals 2 and 3, respectively, needed for CD8 T cell priming and activation. Prior infection may also augment CD8 T cell responses by altering the magnitude of signals 2 and 3 during priming. The accumulated boost in these signals may decrease the remaining activation signal required from signal 1, effectively reducing the threshold of activation for T cells, resulting in inclusion of lower avidity clones and increasing the diversity of the active CD8 T cell repertoire (Souquette and Thomas, 2018). For example, mice co-infected with latent murine gammaherpesvirus 68 or murine cytomegalovirus (MCMV) exhibit augmented activation of APCs and altered cytokine production following secondary challenge with bacteria or influenza virus (Barton et al., 2007; Saito et al., 2013). Co-infected mice also have enhanced CD8 T cell recruitment and activation, though further study is required to determine this mechanism, as well as the impact on the TCR repertoire (Saito et al., 2013). An independent study of MCMV and influenza co-infection further supports these findings and shows that co-infected mice exhibit higher CD8 T cell responses and decreased viral load (Furman et al., 2015). Moreover, altered inflammatory cytokine levels in the microenvironment can also induce activation of memory CD8 T cells in the absence of cognate antigen, a process termed ‘bystander activation’. Compared to the naive state, memory CD8 T cells have decreased activation signal requirements to the extent that they can become activated by cytokine(s) alone or peptide concentrations as low as 1/50th of that required for stimulation of naive T cells (Welsh and Selin, 2002; Youngblood et al., 2013). Indeed, both mouse and human studies have found that influenza virus infection induces activation and expansion of non-specific memory CD8 T cells (Sandalova et al., 2010; Sckisel et al., 2014). Importantly, this population of T cells acquires effector function that contributes to initial pathogen control and impacts disease outcome. These studies demonstrate how antigen-independent signals, which are key to priming lymphocytes, can be shaped by heterologous infections, and, in turn, modify immunity to subsequent exposures. This further supports the significance of and need for additional research which considers the composite immune profile.

### I_0_ landscape

**Figure 3.**
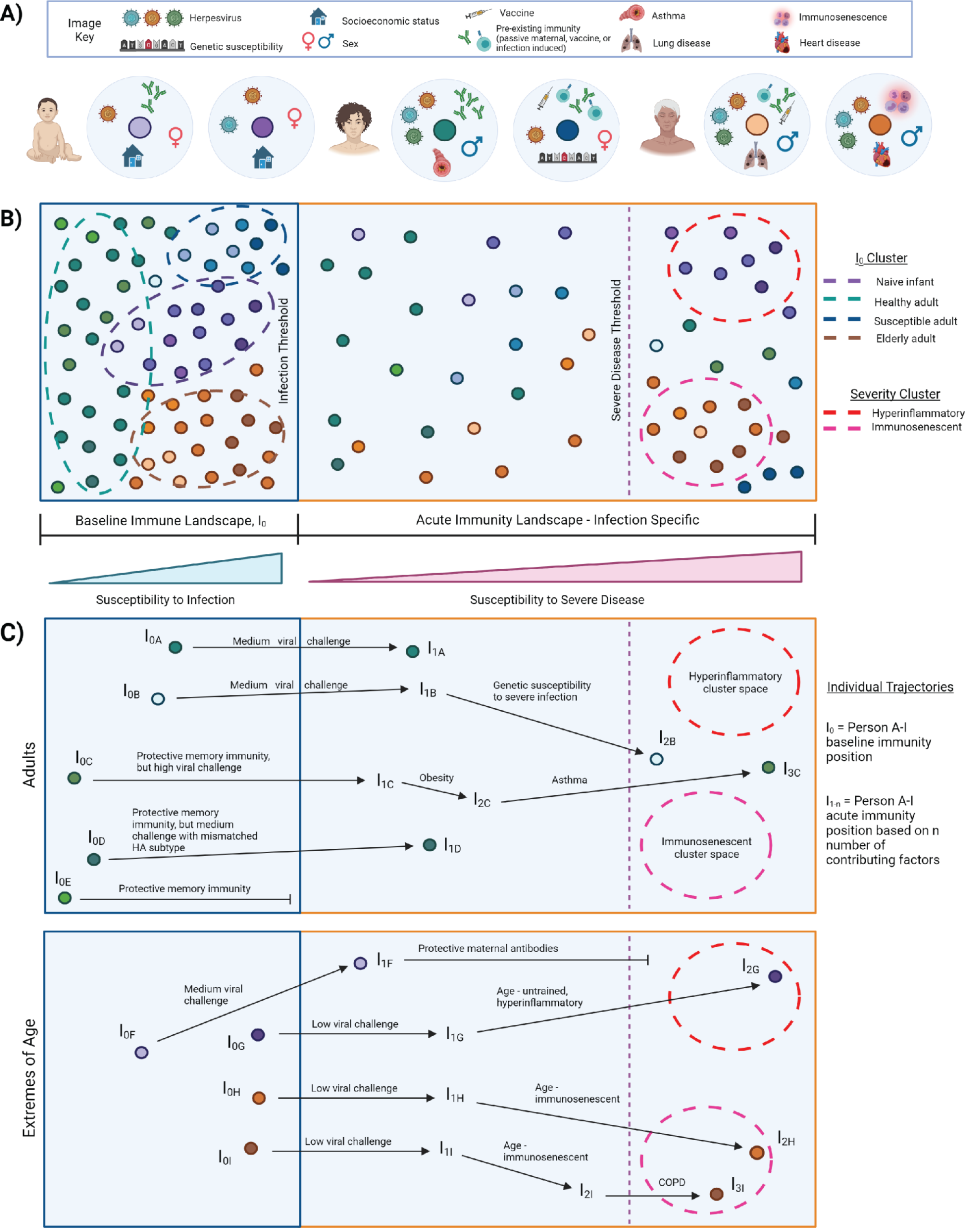
I_0_ and acute influenza infection immune landscape. (**A**) Examples of I_0_ determinant profiles for two infants (purple dots), two adults (blue/green dots), and two elderly subjects, highlighting the vast variation of factors that lead to different positions within the immune landscape. (**B**) Immune landscape during baseline and acute immune states. (**C**) Example trajectories (from panel B) of ‘Adult’ and ‘Extremes of Age’ subjects with varying degrees of influenza disease severity. Arrows correspond to major contributing factors driving a subject’s location within the landscape, and the length of the arrow is associated with the magnitude of the effect of that factor.

I_0_ is the pre-infection, overall state of the immune system, which varies across individuals and changes over time with age, new immune challenge, development of comorbidities, etc. (Figure 3A). Immune variation in I_0_ can be depicted as a multidimensional landscape (Figure 3B), in which subjects with similar I_0_ profiles cluster together. In the context of infectious challenge, there are I_0_ profiles associated with increased susceptibility to infection, and those associated with protection. In the case of influenza, populations with high susceptibility to infection, and thus closer to the threshold of infection, include naive infants (Figure 3B, purple cluster), immunosenescent elderly (Figure 3B, orange cluster), and adults with a pre-existing condition (Figure 3B, blue cluster). I_0_ profiles associated with protection are further from the infection threshold and may represent generally healthy adults or vaccinated subjects (Figure 3B, green cluster). Based on an individual’s I_0_ profile and the amount of pathogen encountered, some subjects will cross the infection threshold and move into the acute immune landscape, which varies by pathogen, and represents the spectrum of disease severity (Figure 3B). An individual’s position within this landscape is also affected by intrinsic and extrinsic host factors, and some subjects will cross the threshold for severe disease.

### I_0_ to acute landscape trajectories

Currently, interpretations of risk factors for severe infectious disease are often very linear. A person becomes infected with a pathogen and each additional risk factor pushes them closer and closer to severe disease - everyone has the same starting point, same ending point, and moves toward that position in the same manner. However, this does not adequately represent the complexity of the immune state and its association with disease severity. Representing immune variation as a landscape (Figure 3B) allows us to move away from linear thinking of risk factor contributions to susceptibility of infection and severe disease. It can represent that two people with similar I_0_ positions, exposed to a medium viral load, would experience a similarly mild infection if all other factors were equal, but one will experience severe disease due to genetic susceptibility (Figure 3C, trajectory A versus B). Additionally, three people may have highly protective memory responses, but only one is protected from infection because the others encountered a high viral challenge, or even a medium viral challenge, but with a novel strain of influenza that has a mismatched HA subtype (Figure 3C, trajectories C–E). In high-risk populations, an infant that would normally be highly susceptible to infection and severe disease is protected by maternal antibodies, and thus is further from the threshold of infection and does not cross the severe disease threshold (Figure 3C, trajectory F versus G). Importantly, the landscape depiction also highlights that the same factor can drive high-risk populations toward severity but in different manners. This is depicted graphically by a single factor altering an individual’s trajectory toward the threshold for severe disease, but the subject’s position within the landscape is driven in different directions and ultimately clusters into different severity immune profiles. For example, the untrained, hyperinflammatory anti-influenza response associated with young age drives an infant toward the upper red severe cluster, whereas severe influenza infection in the elderly is associated with impaired immunity and drives an elderly point toward the bottom-right pink severe cluster (Figure 3C, trajectories G–I). This acknowledges and graphically represents that there may be multiple immune profiles associated with severe infection, which may require different therapeutic options for improved prognosis. Optimum treatment for severe hyperinflammatory infants (Figure 3B, red cluster) will likely be different from that required for severe immunosenescent elderly (Figure 3B, pink cluster). Although not depicted here, it’s important to note the potential for intersections of I_0_ determinants, which may also affect the acute immune response. An ‘intersection’ refers to occasions when determinant A alters the effects of determinant B, either beneficially or detrimentally. The magnitude of the potential impact that arises from such interactions is exemplified in studies that observed impaired trained innate immunity and abrogation of its benefits, sometimes leading to poor prognosis, when an individual has a mutation in proteins involved in key signaling pathways, such as rs2066847 in *NOD2*, and rs3759601 in autophagy gene *ATG2B* (Arts et al., 2015; Buffen et al., 2014; Kleinnijenhuis et al., 2012).

### Implementing I_0_

Thus far, we have described a conceptual framework to relate I_0_ to disease susceptibility and severity. Now, we will outline a mathematical framework to demonstrate how to create an I_0_ landscape, investigate its determinants, and utilize it as an independent variable. The recommended modeling approach is rooted in multivariable linear or logistic regression modeling, depending on whether the outcome variable is continuous or binary, respectively. This facilitates simultaneous assessment of factors that span multiple determinant categories, quantifies the effect of each factor, which allows for comparisons of relative importance, can account for and quantify effects of interactions between factors, and results are easily interpretable (compared to more complex modeling approaches). It is crucial for future studies of human immunity to simultaneously assess multiple determinant categories, because there are contradictions in the literature as to which determinants of immunity are of most importance, and this is likely due, at least in part, to reductive analytical and study design approaches (see ‘Model quality’ section for detailed discussion).

**Figure 4.**
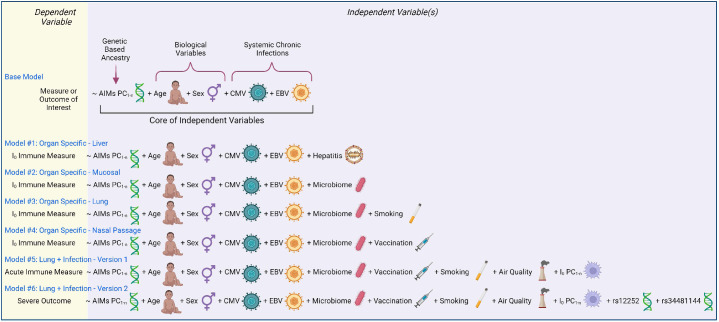
Models of immunity. Examples of statistical models that can be utilized to assess immunity determinants and/or contributions to illness outcome. Models are written in an R programming language format, with the dependent variable to the left of the ‘~’ and all independent variables to the right.

In the proposed framework, whether I_0_ is investigated as a dependent variable or utilized as an independent variable, all models contain a set of ‘core independent factors’ (base model, Figure 4). This includes ancestry informative markers’ (AIMs) principal components, age, sex, CMV status, and EBV status. The selection of core variables is based on an assessment of the literature on immune variation, weighting consideration of results from Patin et al., 2018, because an integrative analytical approach which spanned multiple determinant categories simultaneously was utilized, and found that out of 39 non-genetic determinants, CMV, age, smoking, and sex were the most important non-genetic determinants of basal immune phenotypes, and genetic factors primarily influenced innate cell types, whereas environmental primarily affected adaptive (Patin et al., 2018). It is important to note that under specific experimental contexts or with additional information from newly published studies, this list of ‘core independent factors’ could be expanded. For instance, a study interested in accounting for the effects of human immunodeficiency virus (HIV) by utilizing a cohort comprised of HIV-positive and -negative participants would need to incorporate ‘HIV status’ as a variable. Additionally, HLA haplotype has been associated with susceptibility to and progression of many diseases, including HIV infection, sarcoidosis, ankylosing spondylitis, and asymptomatic coronavirus infection (Augusto et al., 2023; Levin et al., 2015; Schneidewind et al., 2007; Yang et al., 2022). Research aiming to investigate immunity for such illnesses should incorporate HLA into their models. An example of a scientific advancement which may alter the ‘core independent factors’ is if a research study discovered that human papillomavirus, or a similarly common chronic infection, altered the basal immune state, in which case an individual’s serostatus, antibody titer, or other viral-associated metric should be included.

### Creating the I_0_ landscape

Any baseline immune measure could be utilized to generate an I_0_ landscape, but measures should span multiple facets of immunity, and could include non-leukocytes important in initiating or facilitating immune responses, such as fibroblasts or epithelial cells. Notably, there may be immune measures that are only pertinent if select immune pressures or challenges are under investigation. For example, a hemagglutination inhibition (HI) assay titer of 1:40 is associated with a 50% reduction in risk of influenza (Coudeville et al., 2010; Hobson et al., 1972) and would be relevant for an influenza I_0_ landscape, but may not be pertinent for an alternative pathogen- or immune-related illness.

### Utility of I_0_

Once basal immune measures are collected, they can be analyzed as an I_0_ profile by utilizing dimension reduction techniques, such as principal component analysis (PCA). The signatures of individual immune measures that define clusters of interest can then be identified and further investigated to determine optimal therapeutic targets or reagents, and ultimately improve effectiveness of treatment.

Models #1–4 utilize I_0_-related measures as a dependent variable and demonstrate how the base model can be customized to different anatomical locations (Figure 4). Model #1 tailors the base model to a liver-related measurement by including a liver tropic factor - hepatitis infection. Virus status could be binary (yes/no, presence/absence) or continuous (viral load or antibody titer). Model #2 incorporates a factor for the microbiome and would apply to mucosal surfaces, such as the gut or respiratory tract. Model #3 builds on #2 and further customizes the model to the lung by accounting for smoking status. Model #4 adapts #2 and tailors the model to the nasal passage (a site for intranasal vaccination against respiratory infections). Results from these models would provide insight into if and how much a given factor shapes the I_0_ landscape.

Models #5 and 6 utilize I_0_ as independent variables by incorporating an appropriate number of principal component values (PC_1-n_) from the I_0_ landscape. These models are adapted for a lung infection study, and include factors related to susceptibility to infection and pulmonary exposures, such as vaccination and air quality, respectively. Model #5 utilizes an acute immune measure as the outcome variable, and would provide insight into how much the basal immune state (I_0_) affects the magnitude and/or quality of the acute immune response. Model #6 utilizes an infectious disease severity measure as the dependent variable, and expands the genetic component to include the genotype of the two aforementioned SNPs with known severity associations during respiratory viral infection. Results from this analysis would inform how much I_0_ affects illness outcome, and how the magnitude of its effect relatively compares to other determinants.

### Investigation and interpretation of I_0_

To this point, we have utilized common approaches to build the conceptual and mathematical framework of I_0_ with the aim to make this review easily accessible to readers of all backgrounds. With this in mind, a benefit of this computational system is its flexibility, and there are alternatives and special considerations which are important to cover.

In the previous section, we provided an example of creating the multidimensional I_0_ landscape based on PCA, however, a different dimensionality reduction technique may be more appropriate for your study. PCA is a linear transformation method, so it assumes variables are continuous and normally distributed. Non-linear approaches may be more appropriate if your dataset includes multimodal data (discrete and continuous variables) or measures with various underlying distributions (such as those from different omics platforms). Examples of non-linear dimension reduction techniques include, but are not limited to, tSNE (t-distributed stochastic neighbor embedding), UMAP (Uniform Manifold Approximation and Projection), and MultiMap (Jain et al., 2021; van der Maaten and Hinton, 2008; McInnes et al., 2018). Additionally, we graphically depicted the I_0_ landscape in two dimensions (Figure 3) for purposes of illustration and more straightforward conceptualization, but I_0_ can include as many dimensions as is necessary to account for sufficient variation in the dataset, hence ‘PC_1-n_’ in Models #5 and 6. An analysis of the amount of variation accounted for by each dimension would be necessary to determine the optimum number of dimensions to include in a model.

**Figure 5.**
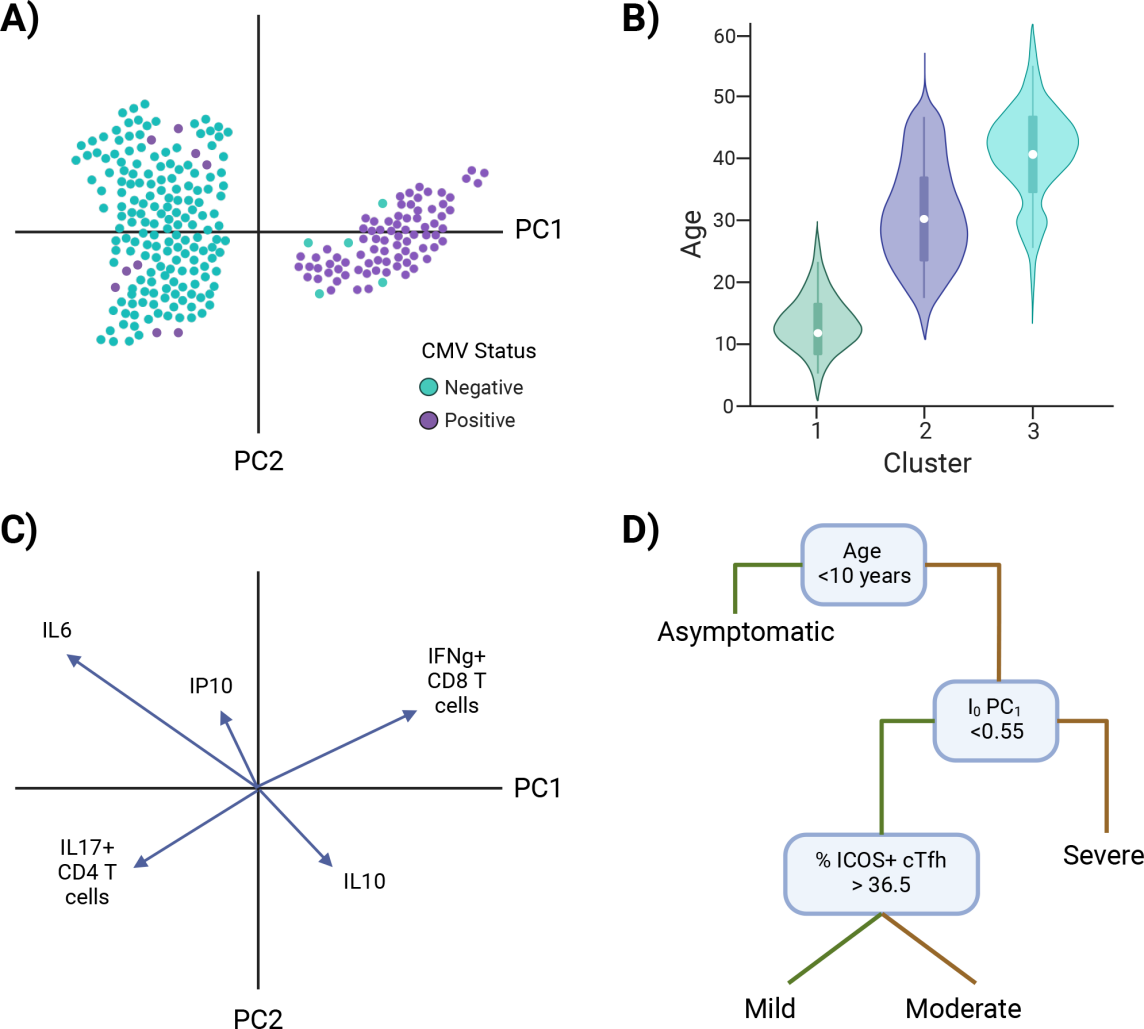
Investigating I_0_. Examples of approaches to identify characteristic features of I_0_ clusters (**A–C**) and infer I_0_ associations with susceptibility to infection or disease severity (**D**). (**A**) Example of overlaying the value of a putative independent variable, cytomegalovirus (CMV) status, on a principal component analysis (PCA) graph. (**B**) Example of subsetting the data by cluster identity and comparing values of a potential defining variable, age. (**C**) Example of a PCA loading plot. (**D**) Example of a decision tree analysis, in which an I_0_ value, PC_1_, is associated with susceptibility to severe disease. Green lines = true, yellow lines = false. *Note: Examples are not related to each other.

Once an I_0_ landscape is created and clusters are identified, there are univariate and multivariate techniques to determine the defining features of I_0_ clusters. Examples of univariate approaches include overlaying, or coloring, points with the value of a putative independent variable (Figure 5A), or subsetting the data based on cluster identity and comparing the values of a potential defining variable (Figure 5B). Examples of approaches that incorporate more than one variable include loading plots and multivariate modeling. Loading plots show a vector for each variable utilized to create a PC graph; each vector is pinned at the origin of PCs and the direction and magnitude of the vector indicate the strength of the effect of a variable on the PC (Figure 5C). Examples of multivariate modeling approaches include, but are not limited to, logistic regression models and random forest modeling, which also provide directionality in results. Similar approaches can be taken to infer how I_0_ relates to susceptibility to infection or severe disease, except I_0_ PCs or clusters are now independent variables and infection status or illness outcome are the dependent variables (Models #5–6 in Figure 4 and Figure 5D). Susceptibility-related outcomes can be discrete or continuous, such as severity category and a participant’s rating of how they felt on a scale of 1–100, respectively.

Studies have shown whether a given factor affects immunity and how much can vary by immune state (basal versus acute), stimulation condition (e.g. poly(I:C) versus lipopolysaccharide), and ethnic population (Allen et al., 2017; Raj et al., 2014; Randolph et al., 2021; Randolph et al., 2021; Souquette et al., 2023). This has several implications when interpreting I_0_ results. First, what affects the basal state may or may not affect the acute state, and vice versa, or the effect may be additive if it affects both the basal and induced acute immune response. Second, I_0_ and acute landscapes are dynamic and pathogen- or stimulation condition-specific, thus immune variation needs to be studied under a variety of immune pressures and caution should be exercised when applying results from one condition to another. Third, it’s critical to study immunity in diverse populations (discussed more in the ‘Model quality’ section). Related to this, it’s important to note that I_0_ is not static, rather it is a ‘snapshot’. An individual’s I_0_ profile will change over time with age, infection history, altered environments, etc. This would be reflected in the I_0_ landscape as shifts in the position of an individual’s profile/point. How much change in a determinant is required for significant perturbation to result in a phenotypically distinct profile is an outstanding question.

Lastly, we have described the I_0_ and acute landscape framework in the context of influenza virus infection, but it can be applied to other medical conditions in which the immune system is a key mediator of illness outcome. However, this approach is limited for diseases with unknown triggers and/or that clinically present at later stages, such as autoimmunity and autoinflammation, because it is difficult, if not impossible, to capture the I_0_ phase (i.e. before illness onset). For conditions that progress to more severe stages over time, a modified framework may be useful, in which the I_0_ and acute phases are replaced with an ‘early/onset’ and ‘chronic’ or ‘late stage’, respectively.

### Model quality

A fundamental concept of modeling is that the output of a model is limited by the quality of the input, and will reflect biases. In the context of translational research, this is crucial to consider for study design, because model inputs are affected by metadata collection, experimental approach, and participant recruitment.

#### Metadata collection

Through utilization of multivariate modeling as an analytical approach, we can computationally account for the effects of multiple factors and compare the relative importance using coefficient estimates from the model summary. However, the thoroughness of the factors included, and thus the model as a whole, is limited by the data that is collected. When assessing, conducting, or collaborating on human immunology research, part of the difficulty in taking a holistic, versus reductionist approach, is in the quality and quantity of metadata collected. There is a small set of basic factors that almost every study includes, such as age and sex, but information gathered beyond this substantially varies. When too sparse, it’s not possible to account for confounding factors, because the data doesn’t exist to incorporate into a model. When too extensive, data are often underutilized, statistical power can be reduced, and time and resources may be wasted. As a field, we need a better understanding of the most influential factors to include in studies of human immunology, referred to here as ‘core metadata’. The set of core metadata variables will likely vary by immune pressure. Thus, deriving a starting list of core metadata will require cross-referencing of corresponding literature, particularly those with integrative analytical approaches, such as Patin et al., 2018, in order to ensure that the most prominent factors are included in metadata collection. Utilization of core metadata does not exclude incorporation of additional variables of interest, rather it would ensure inclusion of factors known to be highly influential on a given outcome of interest, and may aid detection of effects from other factors by accounting for variation attributed to a core metadata variable, which might otherwise contribute to statistical noise (when unaccounted for).

Once known, widespread and consistent incorporation of core metadata into study designs could help improve results’ comparisons across human studies, facilitate better prioritization of resources (both product and staff), and advance our knowledge of complex mechanisms underlying health and disease in human populations.

#### Experimental approach

In addition to consistent metadata collection, more comprehensive assessments of human immunity are needed. To achieve this, measures should account for multiple facets of an immune response, in order to evaluate the overall functional capacity of the immune system in a given state. Ideally, analytes would cover initial detection of pathogens, recruitment of leukocytes, innate effector functions, antigen presentation and co-stimulation to lymphocytes, activation and effector functions of adaptive immunity, repair, and establishment or maintenance of immune memory. Although there are current measures of protection in place, such as the aforementioned HI titers for influenza, there have been substantial technological advancements that facilitate high-throughput, multiparameter assessments which could provide more robust metrics that can be utilized to improve therapeutic strategies, such as vaccination, either through identification of better immune targets or by better assessment of vaccine efficacy. Examples of high-throughput technologies capable of utilizing even small sample amounts include multiplexed, bead-based antibody and cytokine assays that can measure an array of effector functions and facets of immunity (chemotaxis, inflammation, growth factors, etc.), respectively; versatile microfluidics chip platforms, such as Fluidigm, capable of determining gene expression or genotypes for up to 96 targets; high-parameter mass or spectral flow cytometry can examine >35 markers to simultaneously assess innate and adaptive cell populations; and single-cell RNA sequencing, which can be coupled with barcoded antibodies, to investigate the transcriptional and surface marker expression of cell pools.

Whether a dependent or independent variable, data should also be high resolution and, when possible, a clearly defined factor that can be further investigated and validated. For example, the core genetic component in the proposed models is based on AIMs, a curated list of SNPs with varying prevalence across geographic regions. AIM’s chip sizes can range from the low 1000s to over 650,000 SNPs. Utilizing results from dimension reduction analyses, such as AIMs’ principal component values (AIMs PC_1-n_), reduces the factors included in the model, thereby increasing power. Genome-wide association study results are an appropriate ‘high-resolution’ alternative, but AIMs should not be replaced with race/ethnicity, as these categories are ‘low resolution’. Ethnicity is defined by social and cultural norms, whereas race is determined by physical characteristics (Mersha and Abebe, 2015). These categories are dynamic, subjective, and shaped by changing social and geopolitical forces. In contrast, ancestry is fixed and determined by the genetic origin of an individual/population. Non-random mating, often due to geography, can lead to population structure, or systematic differences in allele frequency across subpopulations. This is often reflected in race and ethnic data, as these terms, and their categories, are also associated with nationality/geography.

While informative, careful consideration should be given to their interpretation in clinical and research applications, race and ethnicity categories were not designed for biomedical purposes but rather capture sociological, epidemiological, and biological information, each of which has implications for illness outcome (Malina et al., 2021; Lopez et al., 2021; Mersha and Abebe, 2015). Moreover, their imprecise categorization, which can be overly broad, limited, or overlapping, can lead to inconsistent reporting and results across studies (Borrell, 2005; Malina et al., 2021). This presents a barrier to reducing health disparities by obscuring the root cause, making it difficult to determine whether the most effective intervention is sociopolitical and/or biomedical (Mersha and Abebe, 2015). To aid in proper use of these terms, the National Academies of Sciences, Engineering, and Medicine recently published a consensus study report, ‘Using Population Descriptors in Genetics and Genomics Research’, which discusses the importance of intentional and appropriate use of terminology, explains current approaches, examines best practices based on experimental goals, and provides tools to adopt best practices within the biomedical and scientific communities (National Academies of Sciences Engineering, Medicine, 2023).

#### Study recruitment

Biases in study recruitment, at the basic science and clinical trials level, limit our understanding of health and disease, and, most importantly, impairs the ability to effectively treat patients. Every stage in the process of medical intervention is affected, including screening guidelines, assessment of predisposition, diagnosis, evaluation of disease progression, available therapeutic options, and modulation of dosage to minimize detrimental side effects (Baugh et al., 2022; Drozda et al., 2015; McColley et al., 2023; McGarry and McColley, 2016; National Academies of Sciences Engineering, Medicine, 2022; Watts et al., 2012). Moreover, implementation of tools based on studies with biased demographics, particularly ancestry, can exacerbate health disparities (Carson et al., 1999; Martin et al., 2019). Indeed, recent studies have shown increasing genetic diversity, rather than increasing sample size of individuals of European ancestry, improves identification of novel clinically relevant genetic loci, fine-mapping of functional variants, and portability of polygenic risk scores (Bentley et al., 2019; Cavazos and Witte, 2021; Graham et al., 2021; Wojcik et al., 2019). These studies highlight another important, yet subtle point - the ability to detect effects of a factor hinge on adequate variation in said factor within recruited subjects. This is true for all determinants. For example, studies that aim to determine whether household income is a significant factor should ensure subject recruitment sufficiently spans low, middle, and high incomes. If only middle- to high-income subjects can access the study site, this may lead to a false negative association between household income and the outcome of interest.

Although increasing diversity in study population composition, both genetic and socio-culture, presents statistical challenges, it is imperative in order to: (1) detect influencing factors that may be unique to distinct groups, (2) de-couple correlated variables within populations, (3) understand all pathophysiologies underlying disease, and (4) design broadly applicable, effective therapeutics (De Jager et al., 2015).

### Conclusion

The studies reviewed here provide a framework for defining the I_0_ and acute immune landscapes, which allows for a more comprehensive assessment of immune variation, its determinants, and how this relates to differential outcome. Taken together, it suggests and graphically represents: (1) Variation in I_0_ is associated with distinct immune profiles that vary in functional capacity against a given disease and are associated with different paths to disease severity. (2) Multiple immunological profiles may be associated with severity of a given disease, and more unsupervised approaches are required to determine how many distinct pathophysiologies exist in order to appropriately identify an optimum therapeutic target.

I_0_ has important implications for translational and clinical research as it is commonly conducted to date. First, a ‘one-size-fits-all’ approach will likely be limited in generalizability to the population as a whole, owing to unique immunophenotype groups. Second, equating distinct immune profiles, whether at the I_0_ state for prophylactic treatment or an acute state for therapeutic treatment, may lead to false positive or negative associations, and could explain, at least in part, discrepancies in translating scientific findings at the bench to treatments in the clinic. For example, if a disease has three distinct severity profiles, treatment X may work well for profile A, but this effect is masked, when analyzed collectively, by the lack of effectiveness in profiles B–C. Importantly, skewed outcomes among distinct populations is exacerbated and can go undetected when clinical research lacks diversity in all demographic categories, as populations with varying immune profiles are excluded.

**Figure 6.**
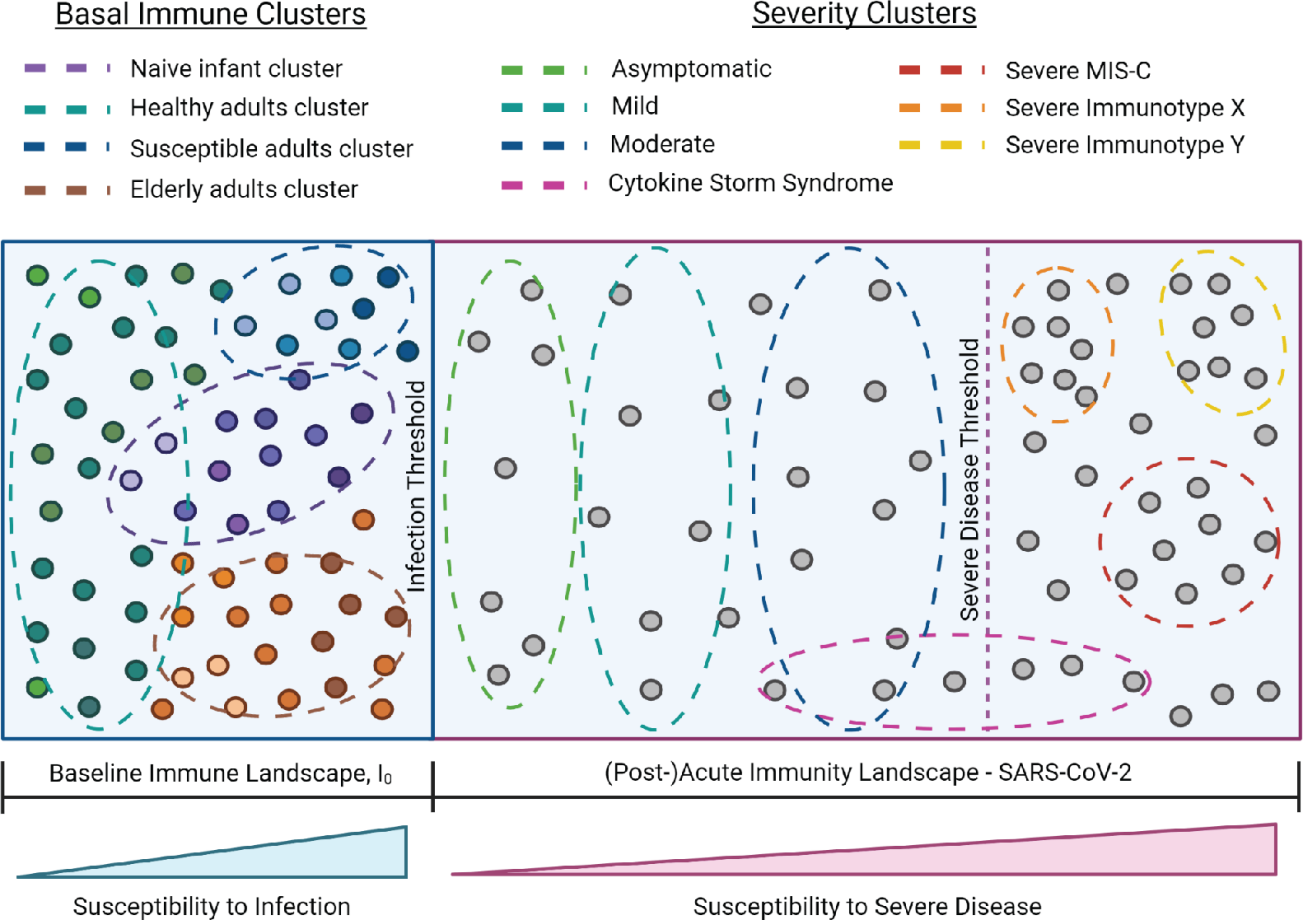
I_0_ and SARS-CoV-2 infection immune landscape. Provisional model of the immunological landscape related to coronavirus disease (COVID). Although more studies are needed to refine specific immune profiles, what we know of the immune response to and presentation of SARS-CoV-2 infection suggests it has a distinct landscape compared to influenza. Mathew, et al. identified three cellular-based immunophenotypes associated with COVID outcome (Mathew et al., 2020). However, it’s unclear how these profiles relate to other immunological signatures of COVID disease presentation. Studies have reported mixed associations between cytokine storm and illness outcome, thus this cluster profile spans the threshold for severe disease (Leisman et al., 2020). Lastly, the acute landscape component includes the post-infectious period in order to incorporate complications such as multisystem inflammatory syndrome in children (MIS-C).

Adopting a conceptual and mathematical framework such as this, as opposed to the traditional linear and supervised approach to examining immunological signatures associated with illness outcome, can aid in the customization of immune-related treatments by distilling many individual immune profiles down to specific clusters that are associated with susceptibility to infection and/or severe disease. Additionally, more studies which comprehensively characterize the immune system as a whole are needed and would provide valuable insight to answer questions such as: To what extent do landscapes vary by pathogen (Figure 6)? Is it possible to determine the total effective number of severity immune profiles for a given infectious disease? If so, how consistent are the main drivers/determinants of severity profiles across different diseases? How consistent are these observations across diverse populations? If inconsistent, is this largely due to genetic factors or other features particular to a given region, such as endemic infectious exposures? How do determinant intersections affect results? The answers to each of these questions will contribute to our understanding of intrinsic and extrinsic host factors that shape the immune system and will have important implications for the development of prophylactics and therapeutics, and clinical decisions on screening and treatment for infectious diseases.
